# Cutaneous Vesicles Caused by Transcutaneous Gas-Monitoring Sensor

**DOI:** 10.4103/0974-2077.53105

**Published:** 2009

**Authors:** Murali Chakravarthy, Sandeep Narayan, Raghav Govindarajan, Subramnyam Rajeev, Vivek Jawali

**Affiliations:** *Chief Consultant Anesthesiologist, Wockhardt Hospitals, Bangalore, Karnataka - 560 076, India. E-mail: mailchakravarthy@gmail.com*

Sir,

Transcutaneous gas monitoring is a noninvasive technique of measurement of oxygen and carbon dioxide tension across the skin. Several studies have validated the accuracy of the values obtained from this monitor.[1,2] Transcutaneous measurements of partial pressure of oxygen and partial pressure of carbon dioxide are based on the principle that a heating element in the electrode elevates the temperature in the underlying tissue. This increases the capillary blood flow, and the partial pressure of oxygen and carbon dioxide, thereby rendering the skin permeable to gas diffusion. The method has been claimed to be safe and free from side-effects. During a study for evaluation of TINA TCPM4 (Radiometer, Copenhagen, Denmark) for transcutaneous carbon dioxide tension and transcutaneous oxygen tension monitoring, two patients developed blister formation due to possible thermal injury. This case report documents the occurrence of such blister formation and the need for awareness amongst physicians about such a side-effect.

**Figure 1:**
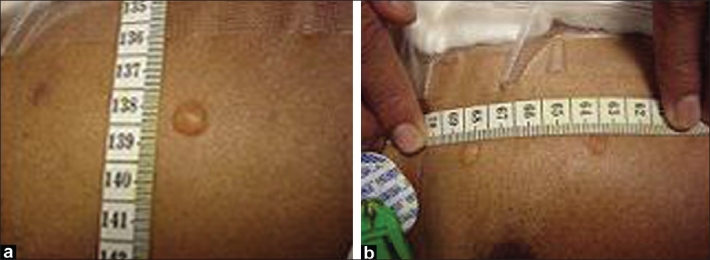
Photographs showing vesicles at the site of transcutaneous gas sensor application

A total of 48 patients who had cardiac surgery during April-July 2008 in our center, underwent transcutaneous gas tension monitoring measured with TINA TCPM4 (Radiometer, Copenhagen, Denmark). During the monitoring, two patients were noticed during routine clinical examination, to have developed vesicles at the site/s of use of the sensor for the equipment on the chest [Figures 1a and b]. There was no previous history of any allergy or atopy in the patients. The first patient developed the vesicle after 3 hours and the second patient after 4 hours of application. In both the patients, the lesions were asymptomatic. There was no history of itching or burning. Dermatological examination revealed tense vesicles on a non-inflammatory base. The lesions subsided spontaneously in 24 hours.

The concept of transcutaneous measurement of respiratory gases looks promising because of its noninvasive nature. The manufacturers, in their product brochure mention the potential for the occurrence of burn injuries at the site of sensor application and recommend the use of sensor at 44°C to minimize the potential risk of burn injury and pressure-induced necrosis. In two of our patients, the application of probes led to blister formation despite adhering to these instructions. The possibility of contact dermatitis was ruled out by the non-inflammatory nature, absence of itching and the occurrence of blisters within 4 hours of application of the probes. Possible mechanisms of blister formation in the patients were thermal injury caused by the increased local skin temperature and the negative pressure within the well of the sensor while the gas expelled via the skin is sampled.

In the light of our experience, we recommend that clinicians wishing to use the transcutaneous measurements may need to restrict the usage time of sensor at any site for less than 3 hours and at temperatures less than 44°C. The sensors need to be applied on aesthetically less significant areas. Further, the possibility of developing such vesicles may be informed to patients during informed consent.
